# Memory matching features bias the ensemble perception of facial identity

**DOI:** 10.3389/fpsyg.2022.1053358

**Published:** 2022-12-01

**Authors:** Tingting Pan, Zheng Zheng, Feiming Li, Jun Wang

**Affiliations:** ^1^Department of Psychology, Zhejiang Normal University, Jinhua, China; ^2^Key Laboratory of Intelligent Education Technology and Application of Zhejiang Province, Zhejiang Normal University, Jinhua, China; ^3^College of Teacher Education, Zhejiang Normal University, Jinhua, China

**Keywords:** ensemble perception, visual working memory, memory matching feature, high-level ensemble, Gestalt principle

## Abstract

**Introduction:**

Humans have the ability to efficiently extract summary statistics (i.e., mean) from a group of similar objects, referred to as ensemble coding. Recent studies have demonstrated that ensemble perception of simple objects is modulated by the visual working memory (VWM) task through matching features in VWM. However, few studies have examined the extending scope of such a matching feature effect and the influence of the organization mode (i.e., the way of combining memory matching features with ensemble properties) on this effect. Two experiments were done to explore these questions.

**Methods:**

We used a dual-task paradigm for both experiments, which included a VWM task and a mean estimation task. Participants were required to adjust a test face to the mean identity face and report whether the irregular objects in a memory probe were identical or different to the studied objects. In Experiment 1, using identity faces as ensemble stimuli, we compared participants’ performances in trials where a subset color matched that of the studied objects to those of trials without color-matching subsets. In Experiment 2, we combined memory matching colors with ensemble properties in common region cues and compared the effect with that of Experiment 1.

**Results:**

Results of Experiments 1 and 2 showed an effect of the VWM task on high-level ensemble perception that was similar to previous studies using a low-level averaging task. However, the combined analysis of Experiments 1 and 2 revealed that memory matching features had less influence on mean estimations when matching features and ensemble properties combined in the common region than when combined as parts of a complete unit.

**Conclusion:**

These findings suggest that the impact of memory matching features is not limited by the level of stimulus feature, but can be impacted by the organization between matching features and ensemble target properties.

## Introduction

Humans have developed a crucial ability called ensemble perception, in which a group of stimulus properties, ranging from low-level features (e.g., orientation, size, and color) to high-level properties (e.g., facial expression, face identity), are rapidly extracted to form summary statistics, such as a mean or a variance of stimuli (Ariely, 2001; Chong and Treisman, 2005; Haberman and Whitney, 2012; Whitney and Yamanashi Leib, 2018). Past studies have shown that this ability has a mutual effect along with visual working memory (VWM) and have found that summary statistics interact with remembered items within the memory representation (Brady and Alvarez, 2011; Bauer, 2017; Corbett, 2017; Corbin and Crawford, 2018; Utochkin and Brady, 2020; Williams et al., 2021). With respect to the impact of ensemble coding on items stored in VWM, discoveries are that estimates of the memorized item were readily biased toward the mean of a subset in the same color as this item (Brady and Alvarez, 2011), or a subset in conformity with Gestalt principles (Corbett, 2017), or a group of homogeneity of items (Utochkin and Brady, 2020). Notably, recent work has focused on the influence of VWM on the process of ensemble coding (Bauer, 2017; Epstein and Emmanouil, 2017; Dodgson and Raymond, 2020; Williams et al., 2021; Jia et al., 2022).

Several papers have explored whether VWM tasks affect ensemble coding and have come to various different ideas of conclusions regarding its impact (Epstein and Emmanouil, 2017; Dodgson and Raymond, 2020; Williams et al., 2021). For example, Epstein and Emmanouil (2017) showed the precision of mean estimations remained when irrelevant items were remembered in a VWM task. However, a few studies have found the bias effect of average estimates emerged when a VWM task (Williams et al., 2021) or a learning task (Dodgson and Raymond, 2020) was done prior to the averaging task and shared features with a part of the stimuli in an ensemble group. Williams et al. (2021) provided an essential explanation for their aforementioned results that VWM influences perceptual averaging through a memory matching feature, while an averaging task without such matching features is unaffected by the memorized object. In the study of Williams et al. (2021), observers were asked to estimate the mean orientation of a stimulus set containing two sets of similar line subsets in different colors while memorizing the colored irregular object. They found that average estimations of all lines were biased toward a subset with the same color as that of the irregular-colored item, suggesting the possibility that the VWM task affected mean estimations through matching features (Williams et al., 2021). However, participants may deliberately devote more attention to memory matching objects and then bias the ensemble estimations. To rule out this possibility, Williams and colleagues compared ensemble biases between a brief duration (i.e., 150 ms) and a long duration (i.e., 500 ms) of the ensemble display in their experiment 3 (Williams et al., 2021). Participants should have reduced bias in brief duration condition because voluntary attentional allocation would be difficult for short presentation durations. The results showed no ensemble bias difference between short and long duration conditions and then excluded the possibility of cuing of attention. Furthermore, the importance of memory matching features in the influence of working memory on ensemble coding was further emphasized by the experiment of Epstein and Emmanouil (2017), who found no connection between the VWM task and the averaging task at the stimulus level without the presence of matching features. Results of their study showed that the accuracy of the size averaging task was unchanged according to levels of working memory load (i.e., remembering zero, two, or four items), nor was it affected by the VWM task. Considering these findings together, we can conclude that the existence of an inter-task shared feature is crucial for the influence of a memorized item on averaging estimations for a group of properties (Epstein and Emmanouil, 2017; Williams et al., 2021). Overall, these findings aligned well with the amplification hypothesis of perceptual averaging (Kanaya et al., 2018), which stated that physically salient elements are involuntarily and automatically weighted more than less salient elements in the contribution of average estimations (Kanaya et al., 2018; Iakovlev and Utochkin, 2021). More importantly, Williams’s study (2021) expanded extension of the amplification hypothesis by showing that memory matching items gained more attentional resources and became more salient compared to nonmatching items, which in turn weighted more in average estimations.

All the aforementioned studies have discussed or confirmed the influence of memory matching features on low-level ensemble coding (Epstein and Emmanouil, 2017; Williams et al., 2021). However, few studies have focused on high-level ensemble coding. Thus, little is known whether the matching feature effect from low-level ensemble coding extends to VWM tasks’ influence on high-level perceptual averaging. As for ensemble perception and object memory, the discrepancy in properties of simple and complex stimuli leads to a hierarchical structure, including both low-level and high-level features (Haberman et al., 2015; Christophel et al., 2017; Ding et al., 2017; Whitney and Yamanashi Leib, 2018). High-level and low-level features differ in their functional roles in our environment (Ding et al., 2017; Whitney and Yamanashi Leib, 2018). Low-level features, such as color, orientation, spatial location, and motion, form a cornerstone for object recognition as well as for taking in an understanding of a scene (Oliva and Torralba, 2006). With respect to high-level features, these are instrumental in offering significant social and emotional information (Cavanagh, 2011; Whitney and Yamanashi Leib, 2018). Having access to ensemble perception of high-level properties is integral to adapting in society, from identifying potential threats to perceiving the emotions of groups of individuals. Furthermore, Haberman et al. (2015) revealed direct evidence that different feature levels might have dissimilar ensemble perception mechanisms, finding that the correlation of summary statistics between high-level (i.e., emotion, face identity) and low-level (i.e., color, orientation) ensemble perceptual tasks was significantly lower than the correlation between those from within the same level. That is, there is not a single, domain-general mechanism supporting all ensemble representation types, and inversely, multiple domain-specific mechanisms work for various feature levels, suggesting a hierarchical structure for levels of ensemble perception. Accordingly, in consideration of this hierarchical structure, matching features through the VWM task would lead to different effects in the high-level ensemble perception. However, this prediction seems contradict to previous literature in which a similar amplification effect driven by physical saliences was evidenced in both low-level (e.g., orientations of lines; Williams et al., 2021) and high-level (e.g., facial expressions; Goldenberg et al., 2021; Goldenberg et al., 2022; Yang and Baek, 2022) ensemble coding. This suggests that the amplification effect is independent of ensemble perception levels. Therefore, we proposed that matching features in VWM would exhibit a similar bias effect on high-level ensemble coding as that on low-level perceptual averaging in Williams’s study (2021).

Additionally, past research has shown that memory matching features are presented as task-irrelevant attributes of ensemble stimuli for the averaging task (Epstein and Emmanouil, 2017; Dodgson and Raymond, 2020; Williams et al., 2021). Thus, it seems that the amplification effect could be driven as long as matching features and averaged properties belong to an object. It is not clear, however, whether having these two types of features belonging to a physical object is essential for the occurrence of the matching feature effect or these two types of features could be perceived as one object based on the Gestalt principle (e.g., common region). It is well-established that Gestalt principles in a bottom-up manner help individual objects to appear together as an integrated unit within VWM, integrating the distributed discrete items into coherent visual information (Xu, 2002; Woodman et al., 2003; Xu, 2006; Xu and Chun, 2007; Hollingworth et al., 2008; Peterson and Berryhill, 2013; Gao et al., 2016; Kalamala et al., 2017; Montoro et al., 2017). In such a visual process, the VWM stored grouped items with Gestalt principles as one object without the single elements (Woodman et al., 2003; Xu and Chun, 2007) and, therefore, elements grouped by Gestalt principles are remembered more easily than elements united without Gestalt principles (Xu, 2002; Woodman et al., 2003; Xu, 2006). For example, Xu (2002) found that the memorization of two properties combined as parts of one object was as effective as remembering two features seemingly belonging to two individual but connected parts whose combination conforms with the Gestalt principle (i.e., connectedness), indicating that the latter combination can be regarded as a complete unit. Based on aforementioned influence of Gestalt principles over VWM, memory matching features and ensemble properties would be united together perceptually with Gestalt principles (i.e., common region), even though physically organized with two individual parts. As a result, it is reasonable to presume that a similar memory matching feature effect would be observed as the condition in which the matching feature stands as a part of the ensemble stimuli.

To test these hypotheses about matching features, and following the experiment of Williams et al. (2021), we used a dual-task paradigm composed of a VWM task and a mean estimation task. In each trial of Experiments 1 and 2, participants were asked to memorize one colored object in a memory display, and then to estimate the mean of the averaging task when remembering the colored object’s properties. Additionally, a common color between the memorized object and a subset of the ensemble display was set up as the memory matching feature. Numerous studies have demonstrated that the color can be the basis of a grouping principle and help to form the hierarchical structure of ensemble representations, allowing for better observation of the bias effect of mean estimations if the memory matching feature functions (Brady and Alvarez, 2011; Luo and Zhao, 2018). The purpose of Experiment 1 was to explore whether the VWM task would influence high-level ensemble coding through the inter-task common feature. We selected face stimuli as the high-level ensemble materials. Face stimuli contain many high-level features used as stimulus properties for the averaging task, such as face similarity (Peng et al., 2021), face identity (Haberman and Whitney, 2009; Bai et al., 2015), and facial expression (Haberman and Whitney, 2007). Among these properties, facial identity was an appropriate choice for the perceptual averaging for our experiments and could be scaled to a 360°circular space. To combine color features (i.e., memory matching features) and ensemble properties belonging to an object, the facial skin color was also considered as the task-irrelevant property matching the color with the VWM object. In addition, in order to make the biasing dimension (i.e., the color of faces) independent of the estimation-task dimension (i.e., the identity of faces) as in previous studies (Iakovlev and Utochkin, 2021; Williams et al., 2021), the ensemble display set was divided into two facial-identity subsets with the mean of each subset either clockwise or counterclockwise from the global mean in circular identity space. Then we manipulated the memorized color to be matched with one of the two subsets (clockwise or counterclockwise), or mismatched with neither subset. Consequently, an amplification effect would be manifested if the estimated mean was biased toward the mean of either clockwise or counterclockwise subset depending on the memory matching color. In Experiment 2, the crucial manipulation was to transform an object which combined the matching feature and the ensemble property as two individual parts with physically separated features, but regarded as a unit with a common region, in such case, connected to each other within an identical space. More specifically, grayed-out faces with the identity information were placed inside boxes colored the same as that of the memorized individual. We predicted a strong bias effect on mean identity estimations, as seen in previous findings, even though matching features were physically separated from the identity face. Furthermore, we expected that our findings would support the assumption that Gestalt principles could be applied to the effect of the VWM on ensemble perception.

## Experiment 1

### Method

#### Participants

Forty-two college students from Zhejiang Normal University were recruited for this experiment and given financial compensation for their participation. Five participants were excluded due to poor performances on the mean identity estimation task (i.e., overall bias <2.5 standard deviations below the group mean) and one participant for poor performance on the VWM task (i.e., overall accuracy <50% below guess rate). The final sample comprised 36 college students (four men; *M* = 20.14 years, *SD* = 1.85; age range = 18–25 years). All participants were right-handed and had normal or corrected vision.

The sample size was determined by an *a priori* power analysis using G*Power software (Version 3.1) with a 0.05 criterion of statistical significance, power of 0.90, a 0.5 correlation between repeated measures, and an effect size (*f*) of 0.2. We used a conservative effect size of 0.25 because different and more complicated stimuli (i.e., face identity) were used in the present study.

#### Stimuli and apparatus

All stimuli were generated using MATLAB (MathWorks, Inc., Natick, MA, United States) with the Psychophysics Toolbox (Version 3 extension) and presented on a 21-inch LCD monitor with a resolution of 1,920 × 1,080 pixels and a refresh frequency of 60 Hz. All stimuli were shown on a uniform black background (RGB = 0,0,0). Each participant sat approximately 57 cm away from the computer monitor with their heads on a desk-mounted chin rest. At this viewing distance, 1° of the visual angle on the display was approximately 36.25 pixels.

The experiment consisted of the irregular object memory task (i.e., the VWM task) and the mean identity estimation task. For each of the stimuli presented in a stimulus display, there were six distinctly separated colors selected from the RGB color space (i.e., blue = 63,108,151; purple = 142,80,141; red = 151,61,87; brown = 148,85,47; olive drab = 102,110,52; dark green = 57,114,105). In the VWM task, stimuli were irregularly shaped 2D objects generated for each trial according to the following restricted conditions. Following the experiment of Cohen and Singh (2007), first, irregular objects were constructed with 12 evenly spaced angles from 1 to 360°. These angles were then used to generate the irregular object’s vertices at random distances subtending 1.1° to 2.2° from the center.

**Figure 1:**
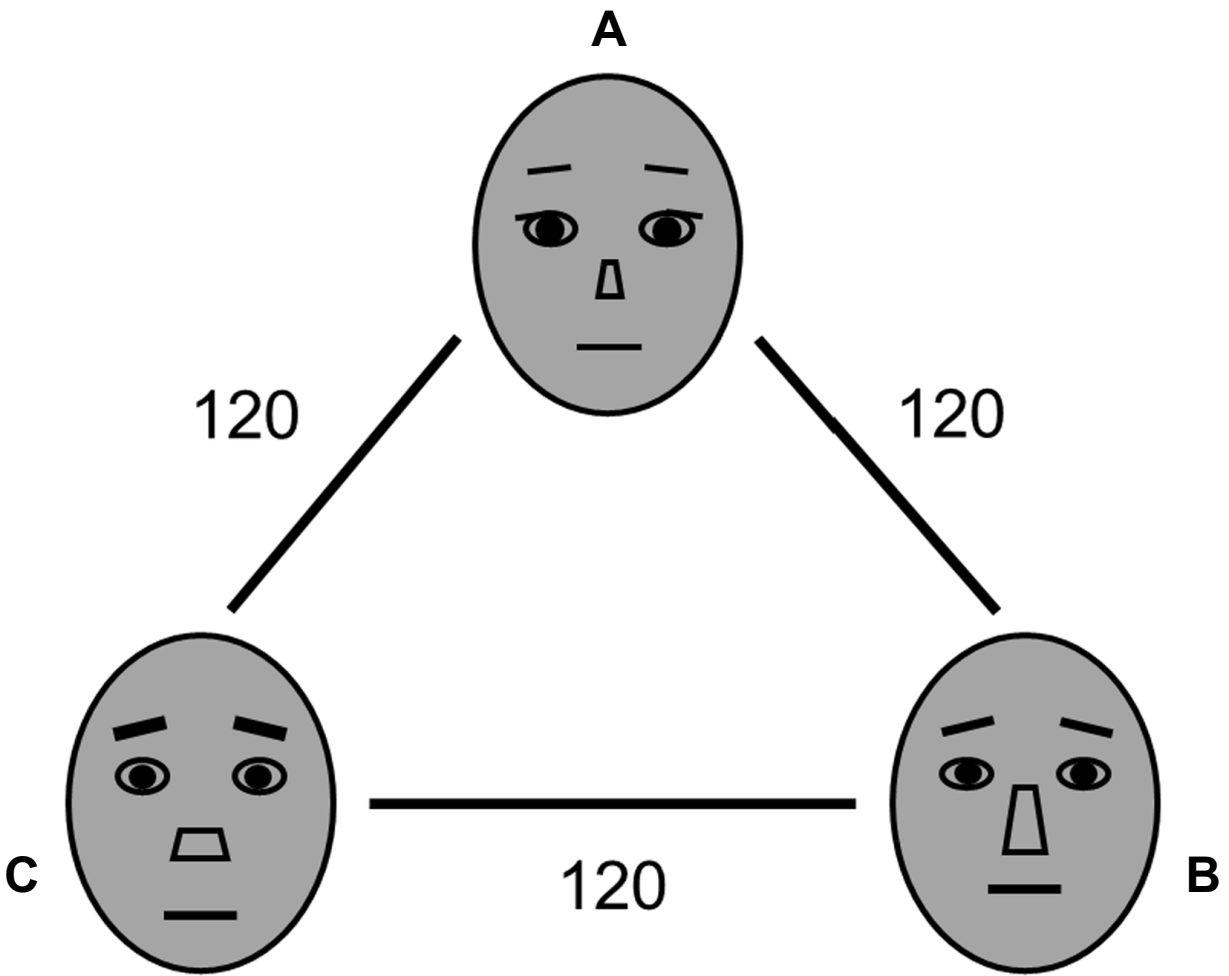
Three neutral female faces **(A–C)** from the NimStim Set of Facial Expressions used to produce 360 face morphs in each color for Experiments 1 and 2, and represented with schematics in figure.

In the mean identity estimation task, four colored face morphs were presented in an ensemble display and 1 gray face was presented in an adjustment display (i.e., ensemble probe). The face morphs comprised a set of 360 face identities for each predefined color and in grayscale by morphing (MorphAge 3.0; Abrosoft Software Corporation) among three distinct neutral female faces from the NimStim Set of Facial Expressions (Tottenham et al., 2009), which are represented with schematics in Figure 1 (A-B-C-A). The face identity of these face morphs were circular stimulus spaces with 360°. To minimize the difference in the faces’ physical features, the face morphs were scaled with luminance normalized using the SHINE toolbox (Dal Ben, 2019) in MATLAB.

In the ensemble display, four face morphs were each subtended 4.30° × 6.00° of the visual angle, occupying each quadrant 5.18° from the fixation point (2 × 2 grid). The entire set of four faces was separated into two distinct equally-numbered identity subsets by two predefined colors around the mean identity of the whole set (−36, −12, +12, +36 relative to the global mean of the four faces). The identity values of one subset were clockwise to the global mean while those of the other subset were counterclockwise to the global mean. In an adjustment display, a randomly selected gray face from the 360 identities, and was subtended at 4.30° × 6.00° of visual angle at the center.

#### Procedure

The VWM task and mean identity estimation task were combined in a single trial. Participants were asked to study the form or color of an irregularly-shaped 2D object for the VWM task and to report the mean identity of a set of four faces for the mean estimation task. The experiment consisted of matching and mismatching trials. The irregular object’s color in the VWM task matched the color of the clockwise subset for half of the matching trials (i.e., clockwise matching condition, CM) while the color of the VWM object matched that of the counter-clockwise subset for the other half of the matching trials (i.e., counter-clockwise matching condition, CCM). In the mismatching trials, neither of the two subset colors matched the color of the VWM object (i.e., mismatching condition, MM).

**Figure 2:**
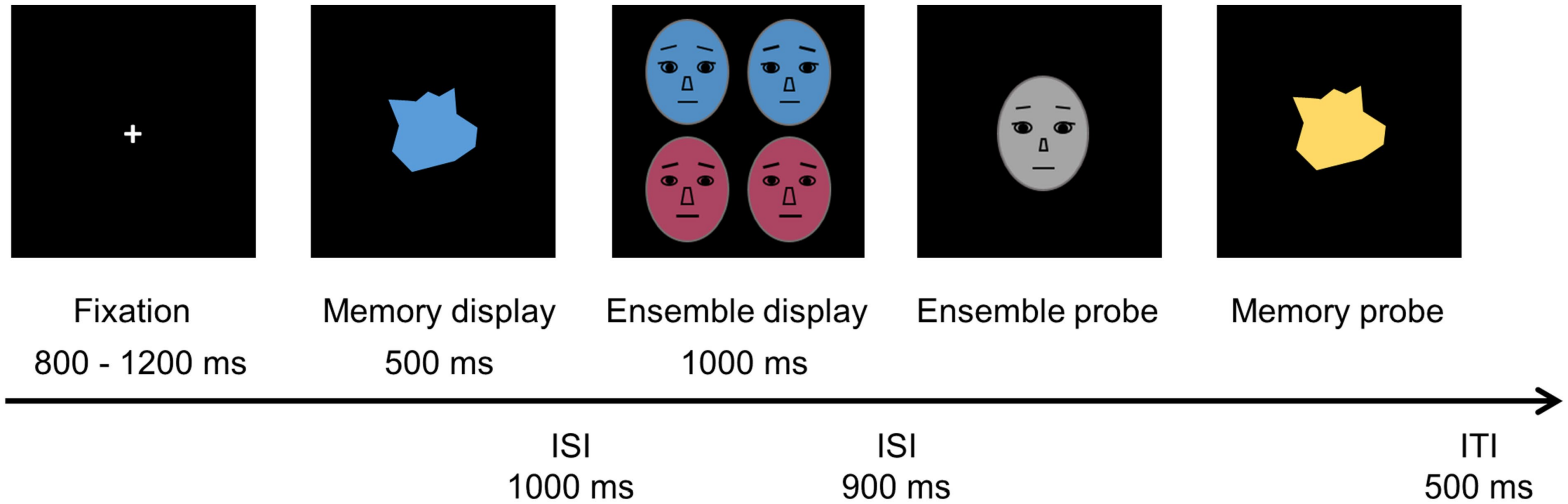
Sequence of display in Experiment 1.

Figure 2 illustrates the procedure of a single trial and identity faces shown in the figure were represented with schematics. Each trial began with a fixation central cross (0.5° × 0.5°) presented for a randomly varied interval of 800 to 1,200 ms. A memory display then followed, presented for 500 ms. Participants were asked to memorize the form and color of the irregularly-shaped object positioned at the center of the display. The color of the VWM object was randomly selected from the predefined colors. After a 1,000-ms blank screen, a set of four faces appeared in an ensemble display for 1,000 ms. Following a 900-ms blank interval, a gray face with a random identity selected from the 360 possible facial identities appeared centrally in an ensemble display. Participants were asked to place the face in a perceived mean of an identity-face set by pressing the “left” or “right” arrow keys to tilt the face clockwise or counter-clockwise in the identity space, and to press the “space” key to lock the estimated identity as their answer. After the mean identity adjustment, recall of the studied individual item was tested. Participants were asked to judge whether the second object displayed in a memory probe was identical to or different from the object studied in the VWM display of the trial and to report their answer by pressing the “S” (same) or “D” (different) keys. The same trials and different trials occurred at an equal frequency. For different trials, either the color or the form could differ from the studied object (but both would never change simultaneously) with both options occurring at an equal frequency. Once the participant gave their answer by pressing the relevant key, the trial ended with a 500 ms blank inter-trial interval. Participants were instructed to be as accurate and as quick as possible, but there was no time limit for their response.

Prior to starting the experiment, all participants completed a practice block consisting of 12 trials (four trials from each condition) to familiarize them with the VWM and identity estimation tasks. The experiment comprised six blocks, each composed of 36 trials with an equal trial number for each condition, with a short break after every second block.

#### Data analysis

The purpose of both Experiments 1 and 2 was to measure whether mean identity estimations would be affected by the shared color of a matching subset when a colored irregular object was held in VWM, and further to explore the extending scope and organization mode of feature-matching. First, we analyzed the accuracy of the VWM task across three matching conditions (i.e., CM, CCM, MM), measured as the proportion of correct responses to the VWM object question (i.e., VWM accuracy). Analyses of mean identity estimations were limited to trials in which participants correctly recalled the first colored object that had been displayed in the VWM display. Following previous studies (Iakovlev and Utochkin, 2021; Williams et al., 2021), in each trial, we calculated response errors measured as the smallest difference between the participant’s selected face in an ensemble probe and the actual global mean (Response error = estimate response - actual mean) in the circular space of face identities. The resulted error distribution across trials were further employed to estimate two important indicators of ensemble perception *via* the CircStat toolbox using MATLAB (Bays et al., 2009; Berens, 2009): the circular standard deviation (CSD) of error distribution as the estimated precision and the mean of error distribution (ensemble bias) as the tendency of the estimated mean. Accordingly, the positive bias would reflect a tendency to estimate the mean value toward the mean of a clockwise subset while the negative bias would reflect a tendency to estimate the mean value toward the mean of a counterclockwise subset. Participants were excluded if their proportion of correct responses in the VWM task and/or response errors in the mean identity estimation task were greater or lower than 2.5 *SD*s above the overall mean. Participants’ trials were excluded if their performance in the VWM task and/or the mean identity estimation task was greater or lower than 2.5 *SD*s.

Lastly, classical and Bayesian statistical analyses were conducted using Jamovi (Version 2.2.5.0; The Jamovi Project, 2021) developed by R (retrieved from https://www.jamovi.org) and JASP (Version 0.10.0.0; The JASP Team, 2022). We employed within-subject one-way analyses of variance (ANOVAs) to compare the performances in the three matching conditions of VWM accuracy, CSD, and bias, and then employed the Bonferroni correction to compare these conditions with each other.

### Results and discussion

#### Visual working memory accuracy

**Figure 3:**
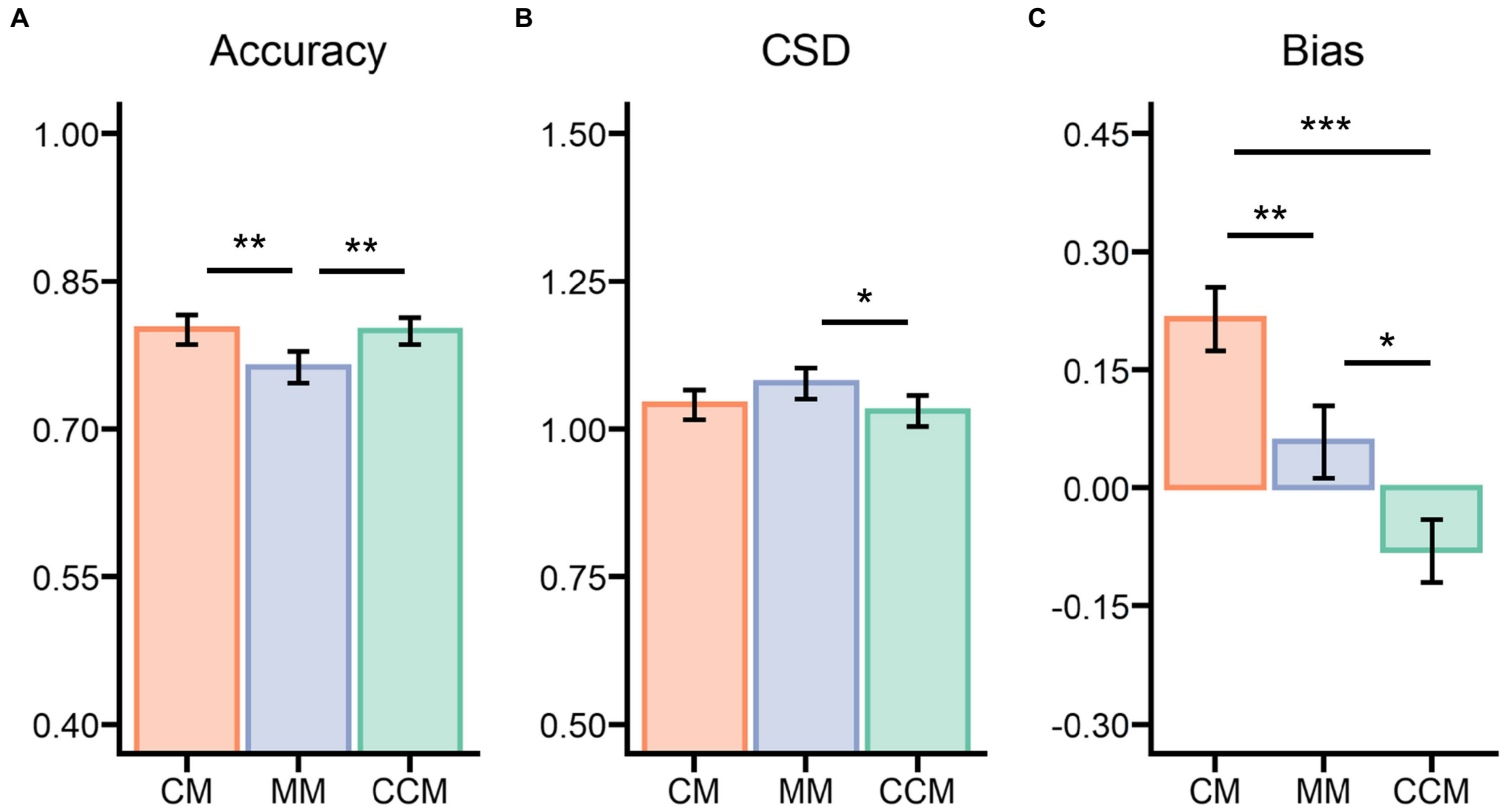
Results of Experiment 1 for the three matching conditions on **(A)** VWM accuracy, **(B)** the ensemble circular standard deviation (CSD) of the mean identity estimations, and **(C)** the ensemble bias parameter of the mean identity estimations. CM, clockwise matching condition; MM, mismatching condition or control condition; CCM, counterclockwise condition. *p < 0.05, **p < 0.01, ***p < 0.001.

The studied irregular objects were recalled correctly and judged accurately on 78.74% of the trials (*M* = 0.787, *SE* = 0.013, ranging from 0.588 to 0.935). The performance on the VWM task differed significantly between the CM, CCM, and MM conditions (see Figure 3A), *F*(2,70) = 8.069, *p* < 0.001, η_p_^2^ = 0.187, BF_inclusion_ = 40.981. The accuracy of the control condition (*M* = 0.763, *SE* = 0.016) was lower compared to the CM condition (*M* = 0.801, *SE* = 0.015), *t*(35) = 3.538, *p* = 0.002, Cohen’s *d* = 0.431, and the CCM condition (*M* = 0.800, *SE* = 0.013), *t*(35) = 3.417, *p* = 0.003, Cohen’s *d* = 0.416. Meanwhile, the accuracy of the VWM task showed no difference between the CM and CCM conditions, *t*(35) = 0.121, *p* = 1.000, Cohen’s *d* = 0.015.

#### Ensemble circular standard deviation

For the mean identity task, there was a significant difference on the ensemble CSD among the matching conditions (see Figure 3B), *F*(2,70) = 4.040, *p* = 0.022, η_p_^2^ = 0.103, BF_inclusion_ = 2.049. Specifically, there was greater variance in the error distribution for the control condition (*M* = 1.078, *SE* = 0.026) in comparison to the CCM condition (*M* = 1.031, *SE* = 0.026), *t*(35) = 2.715, *p* = 0.025, Cohen’s *d* = 0.304, but not compared to the CM condition (*M* = 1.042, *SE* = 0.025), *t*(35) = 2.087, *p* = 0.122, Cohen’s *d* = 0.233. There was no significant difference in error distribution between the CM and CCM conditions, *t*(35) = 0.628, *p* = 1.000, Cohen’s *d* = 0.070.

#### Ensemble bias

The critical question was, *What happens to mean identity estimation when participants have an accurate representation of the studied object in the VWM*? After calculating the bias parameter, participants were found to perform significantly differently across the different conditions (see Figure 3C), *F*(2,70) = 16.47, *p* < 0.001, η_p_^2^ = 0.320, BF_inclusion_ = 24792.156. They were more precise at estimating the mean in the control condition (*M* = 0.058, *SE* = 0.046) when subset colors mismatched the color of the studied VWM object than in the other matching conditions (i.e., CM, CCM) wherein the subset color matched that of the irregular object. Mean estimations of the control condition trials nearly matched the global mean, *t*(35) = 1.270, *p* = 0.213, Cohen’s *d* = 0.21, BF_10_ = 0.375. Compared with the control condition, both the CM (*M* = 0.214, *SE* = 0.040), *t*(35) = 3.045, *p* = 0.010, Cohen’s *d* = 0.619, and CCM (*M* = −0.079, *SE* = 0.040), *t*(35) = 2.691, *p* = 0.027, Cohen’s *d* = 0.547, conditions showed a bias toward the mean of a matching subset (local mean), and there was a significant difference in the bias parameter between these two conditions, *t*(35) = 5.736, *p* < 0.001, Cohen’s *d* = 1.166.

Overall, our data and the comparisons between the three matching conditions indicated that there was a significant effect of the memorized objects studied in the VWM task on the mean identity estimations. Performances in the VWM task and the mean estimation task were consistent with the findings of previous studies on the effect of a low stimulus level on the ensemble coding task. Therefore, the influence of the matching feature does extend from low-level ensemble coding to high-level ensemble perception, suggesting that the inter-task shared feature effect is not limited by the stimulus feature level. As expected, although different ensemble levels might affect the precision and efficiency of summary statistics with distinctive visual processes (Haberman et al., 2015), our findings suggest that the amplification effect caused by memory matching features is independent of ensemble perception levels, and different ensemble levels might be identical in terms of their pattern of information storage and extraction. Additionally, we also found that the recall precision of a studied object improved due to the memory matching feature. This is consistent with previous studies showing that perceptual averaging influenced the representation of individual items in VWM (Brady and Alvarez, 2011; Corbett, 2017; Utochkin and Brady, 2020). For example, Brady and Alvarez (2011) found that the reported size of the memorized circle was biased toward the mean size of previously presented circles that matched the memorized circle in color.

## Experiment 2

### Method

#### Participants

Thirty-eight college students from Zhejiang Normal University were recruited for this experiment and received financial compensation for their participation. Two participants were excluded due to poor performances on the mean identity estimation (i.e., overall bias <2.5 standard deviations below the group mean). Therefore, the final sample contained 36 participants (8 men; *M* = 21.72 years, *SD* = 2.44), and a sample size that was the same as that of Experiment 1. Selection criteria and procedure was the same as in Experiment 1. As in Experiment 1, all participants were right-handed and had normal or corrected vision.

#### Stimuli and procedure

**Figure 4:**
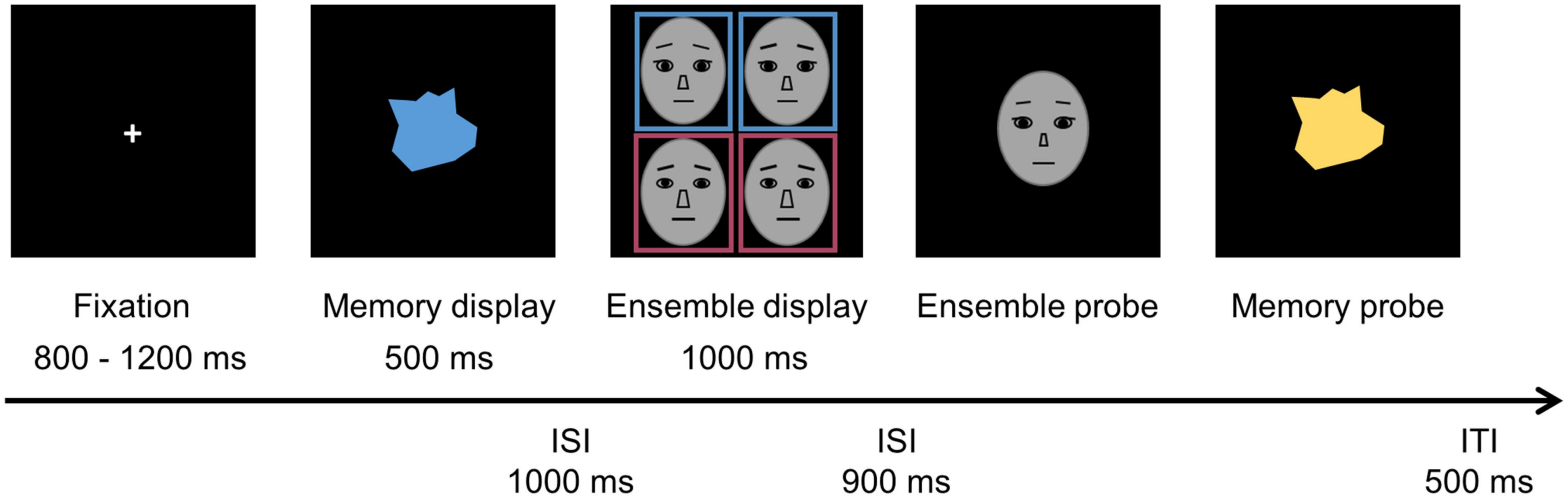
Sequence of the display in Experiment 2, with the identical procedure and task requirements as Experiment 1.

As shown in Figure 4, the task used in Experiment 2 was identical to that of Experiment 1, except that the stimuli in this experiment were identity face morphs in grayscale presented in a colored box in the common region, rather than being face morphs in a colored mask. Each color box subtended 7.15° × 7.50° of the visual angle with the outlines of 0.14° width. A grayscale face was positioned in the center of each color box with a quadrant 5.18° from fixation. An ensemble display contained four items distributed equally in a set. Participants were asked to complete the task in the same manner as in Experiment 1.

### Results and discussion

#### Visual working memory accuracy and ensemble circular standard deviation

**Figure 5:**
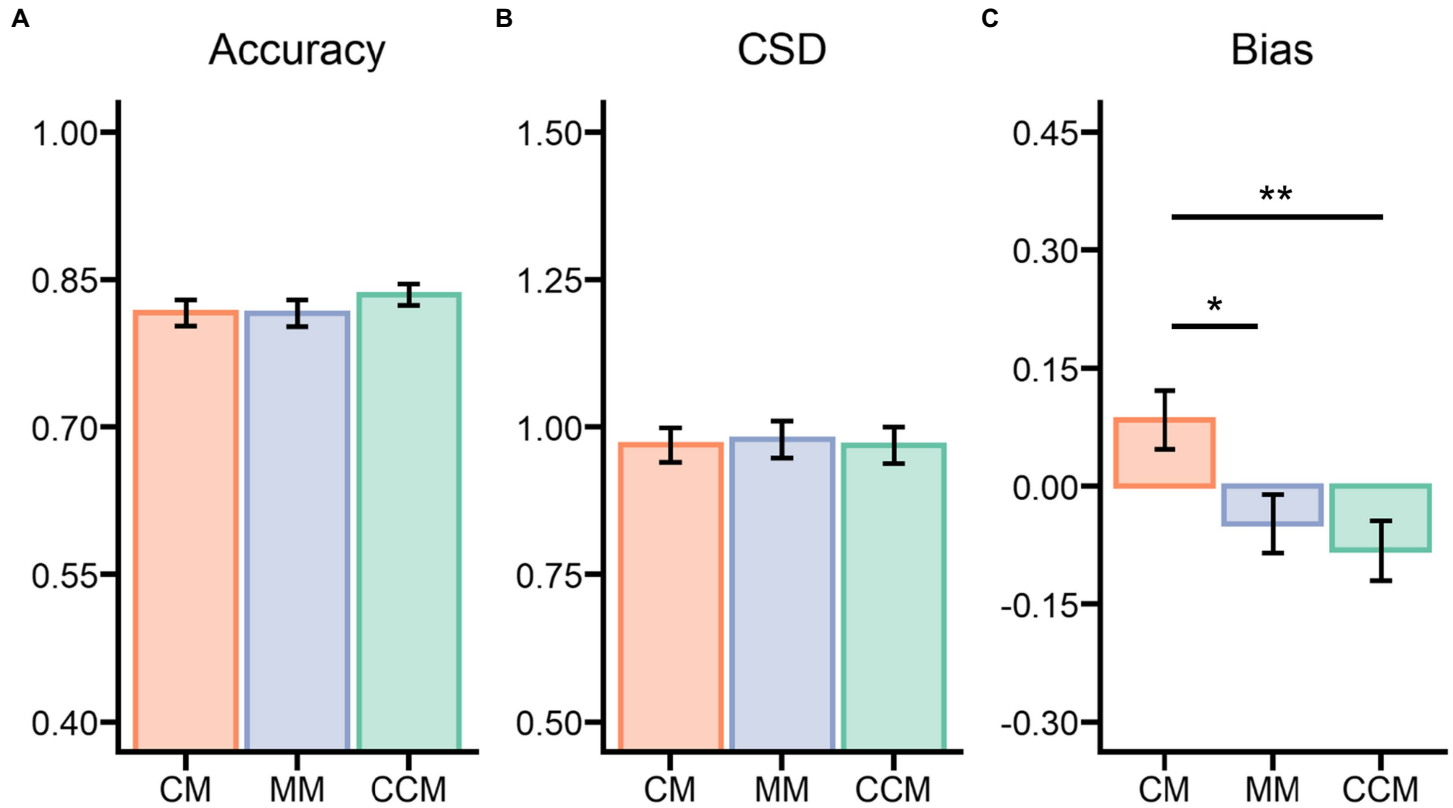
Results of Experiment 2 for the matching conditions on **(A)** VWM accuracy, **(B)** the ensemble circular standard deviation (CSD) of the mean identity estimations, and **(C)** the ensemble bias of the mean identity estimations. CM, clockwise matching condition; MM, mismatching condition; CCM, counterclockwise condition. *p < 0.05, **p < 0.01.

The studied irregular items were recalled correctly and judged accurately on 82.23% of the trials (*M* = 0.822, *SE* = 0.011, ranging from 0.681 to 0.954). In the three matching conditions, performance on the VWM task differed insignificantly (see Figure 5A), *F*(2,70) = 2.356, *p* = 0.102, η_p_^2^ = 0.063, BF_10_ = 0.565. Furthermore, no significant difference was found in CSD in the matching conditions (see Figure 5B), *F*(2,70) = 0.271, *p* = 0.763, η_p_^2^ = 0.008, BF_10_ = 0.106.

#### Ensemble bias

We tested for any matching feature effect on the bias parameter when facial items and matching colors were combined using the Gestalt principle of common region. Results showed that the main effect of the bias parameter was significant in the matching conditions (see Figure 5C), *F*(2,70) = 5.915, *p* = 0.004, η_p_^2^ = 0.145, BF_inclusion_ = 15.071. The CM (*M* = 0.084, *SE* = 0.037) and CCM (*M* = −0.082, *SE* = 0.039) conditions showed bias toward a matching subset mean (local mean) and differed from one another, *t*(35) = 3.257, *p* = 0.005, Cohen’s *d* = 0.732. However, estimates of the control condition (*M* = −0.048, *SE* = 0.037) were lower than those of the CM condition, *t*(35) = 2.587, *p* = 0.035, Cohen’s *d* = 0.581, but were close to those of the CCM condition, *t*(35) = 0.670, *p* = 1.000, Cohen’s *d* = 0.151.

## Combined analysis of Experiment 1 and Experiment 2

**Figure 6:**
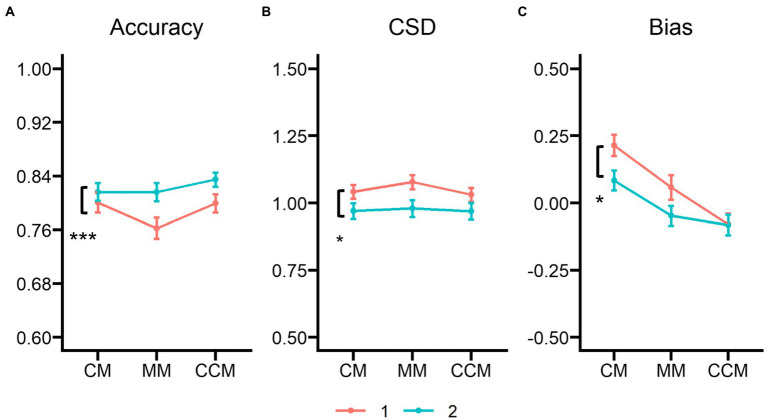
Results of the combined analysis of Experiments 1 and 2 for the matching conditions on **(A)** VWM accuracy, **(B)** the ensemble circular standard deviation (CSD) of the mean identity estimations, and **(C)** the ensemble bias parameter of the mean identity estimations. CM, clockwise matching condition; MM, mismatching condition; CCM, counterclockwise condition. *p < 0.05, ***p < 0.001.

We explored whether there was a significant effect between organization according to Gestalt principles (Experiment 2) and the combination of matching colors and facial identities on an identical face (Experiment 1). We tested whether there would be differences in VWM accuracy, ensemble CSD, and bias between Experiments 1 and 2. As such, we used 2 (experiment: Experiment 1 vs. Experiment 2) × 3 (matching condition: CM vs. CCM vs. MM) repeated measures ANOVAs on these parameters, and Figure 6 show these comparison between Experiment 1 and Experiment 2.

### Visual working memory accuracy

Results showed a small main effect of experiment, *F*(1,70) = 4.005, *p* = 0.049, η_p_^2^ = 0.054, BF_inclusion_ = 2.426, with higher accuracy in Experiment 1 (*M* = 0.788, *SE* = 0.012) than in Experiment 2 (*M* = 0.822, *SE* = 0.012). The main effect of the matching condition was significant, *F*(2,140) = 7.554, *p* < 0.001, η_p_^2^ = 0.097, BF_inclusion_ = 31.154. Participants remembered less accurately in the control condition (*M* = 0.789, *SE* = 0.011) than in the CM condition (*M* = 0.809, *SE* = 0.010), *t*(70) = 2.621, *p* = 0.029, Cohen’s *d* = 0.235, and the CCM condition (*M* = 0.817, *SE* = 0.009), *t*(70) = −3.796, *p* < 0.001, Cohen’s *d* = −0.340. No difference was found between the CCM and CM conditions, *t*(70) = −1.175, *p* = 0.726, Cohen’s *d* = −0.105. The interaction of experiment and matching condition was also significant, *F*(2, 140) = 3.338, *p* = 0.038, η_p_^2^ = 0.046, BF_inclusion_ = 3.114, but there was no significant comparison found across CM, CCM, and MM conditions between Experiments 1 and 2, *t*s < −0.652, *p*s > 0.101, Cohens’*d*s < −0.189.

### Ensemble circular standard deviation

On the analysis of the ensemble CSD, there was a significant main effect of experiment, *F*(1,70) = 4.245, *p* = 0.043, η_p_^2^ = 0.057, BF_inclusion_ = 1.251, in which the CSD of Experiment 1 (*M* = 1.050, *SE* = 0.027) seemed higher than in Experiment 2 (*M* = 0.973, *SE* = 0.027). Furthermore, there was a very small main effect of matching condition, *F*(2,140) = 3.353, *p* = 0.038, η_p_^2^ = 0.046, BF_inclusion_ = 0.617, with the higher CSD in the MM condition (*M* = 1.029, *SE* = 0.020) than that in the CCM condition (*M* = 1.000, *SE* = 0.020), *t*(70) = 2.449, *p* = 0.047, Cohens’*d* = 0.170. However, there was no significant experiment × matching condition interaction, *F*(2, 140) = 1.293, *p* = 0.278, η_p_^2^ = 0.018, BF_inclusion_ = 0.270, and insignificant comparisons across the matching conditions, *t*s < 2.464, *p*s > 0.235, Cohen’s *d*s > 0.581.

### Ensemble bias

Finally, and of greatest importance, there was a significant effect found on ensemble bias. The bias parameter differed between Experiment 1 (*M* = 0.064, *SE* = 0.027) and Experiment 2 (*M* = −0.015, *SE* = 0.027), *F*(1,70) = 4.359, *p* = 0.040, η_p_^2^ = 0.059, BF_inclusion_ = 1.101. Specifically, the bias effect of Experiment 1 was more significant than that of Experiment 2. Additionally, there was a difference on the matching conditions, *F*(2,140) = 20.674, *p* < 0.001, η_p_^2^ = 0.228, BF_inclusion_ = 1.535 × 10^6^. Estimates of the CM condition (*M* = 0.149, *SE* = 0.028) were biased more toward a local mean of a matching subset than those of the CCM condition (*M* = −0.081, *SE* = 0.028), *t*(70) = 6.363, *p* < 0.001, Cohen’s *d* = 0.959, and of the MM condition (*M* = 0.005, *SE* = 0.030), *t*(70) = 3.983, *p* < 0.001, Cohen’s *d* = 0.600. There was no difference found for the CCM and MM conditions, *t*(70) = 2.380, *p* = 0.056, Cohen’s *d* = 0.359. However, the interaction of experiment × matching condition was insignificant, *F*(2,140) = 1.769, *p* = 0.174, η_p_^2^ = 0.025, BF_inclusion_ = 0.868. In these analyses, we found the bias effect still was strong in Experiment 2 but weaker than that of Experiment 1.

From these results, we can conclude that the VWM task can affect mean estimations through inter-task shared features when matching colors and ensemble identities are combined according to Gestalt principles. Nonetheless, it is non-negligible that organization modes between matching features and the averaged properties modulated the extent of the memory matching feature effect on the estimated bias of perceptual averaging. In contrast to the stimulus organization in Experiment 1, the combination of the Gestalt principle in Experiment 2 weakened the bias effect of mean identity estimations but improved estimated precise on mean estimations. Moreover, in Experiment 2, the VWM task performance seemed unaffected by the matching color, and the accuracy of the matching conditions improved at an integral level. These results indicate that the effectiveness of the memory matching feature may reduce somewhat when facial identities and matching colors are physically separated, even when integrated according to the Gestalt principle.

## General discussion

Although the impact of VWM on summary statistics was varied (Bauer, 2017; Epstein and Emmanouil, 2017; Williams et al., 2021), memory matching features appeared to play a meaningful and functional role in the influence of a single studied item over average estimations. Based on this, Experiment 1 explored the scope of the matching feature from a low-level feature (i.e., orientation) to a high-level property (i.e., face identity). The results of Experiment 1 developed the findings of Williams et al. (2021), revealing that the influence of the matching feature could extend from mean estimations of low-level orientation to the perceptual averaging process of high-level facial identity, concluding that the memory matching feature effect occurs across all levels of ensemble perceptions. That is, when an irrelevant individual shares common information about stimulus features with the latter averaging task even that is detrimental to the task’s goal, this information led to average estimations which deviated from the summary statistics of a set, but biased toward the mean of a part with the same feature. According to results of Experiment 1, shared features stored in VWM largely explain the bias effect of mean estimations, which can be observed in a wide range of properties.

Experiment 2 verified whether the way stimuli are combined between matching colors and ensemble properties modulated the influence of the memory matching feature on the averaging task. Results showed a similar effect as in Experiment 1, in that estimates were biased toward averages of a subset in the same color as the memorized object if the matching colors were integrated with ensemble identities under the common region cue, but non-overlaid on face stimuli. Such results extended the “matching features” to a broader context regarding the influence of matching feature maintained in VWM over mean estimations. Moreover, the bias effect of mean estimations by features in VWM was unstable and varied according to the specific stimulus integration for the averaging task. Taken together, the results of Experiments 1 and 2 support the notion that VWM tasks can influence average estimations by the matching features, modulated by the stimulus combination between task-irrelevant colors in VWM and ensemble target properties. Our findings provide novel evidence that the impact of VWM on the process of ensemble coding can be explained by matching stimulus features between tasks from the stimulus level, which supports the idea that averaging processing can be guided by top-down memory of a prior task or past experience in the visual field (Dodgson and Raymond, 2020; Talcott and Gaspelin, 2020; Ramgir and Lamy, 2022). In other words, participants remembered the irregular object’s color in a goal-directed manner, and estimates were biased toward a subset in the same color as the studied object by a way of amplifying the properties highlighted by VWM. Previous studies have shown that this way of the averaging phase aligns with the amplification hypothesis, stating that physically salient items are more largely weighted than less salient items in the determination of summary statistics (Kanaya et al., 2018). In our study, memory matching colors led to the amplification effect on mean estimations. Nonetheless, matching feature effect can also be explained by the feature-weighting account (Maljkovic and Nakayama, 1994) or the episodic retrieval model (Thomson and Milliken, 2013), both of which stress the importance of shared features on consecutive phases. Both accounts refer to feature inter-trial priming whereby attention may be automatically captured by the color or positioning remembered from the previous trial, resulting from prior experience (Lamy and Kristjansson, 2013; Ramgir and Lamy, 2022). In the present study, these accounts applied to continuous tasks (i.e., the VWM task and the mean estimation task) with the same color feature. The feature-weight account suggests that the bias effect is due to the fact that estimates are easily biased toward a memory matching subset that becomes more activated due to being shared by the relevant feature from the previous target. Episodic retrieval model supports a lasting influence of shared features, specifically, and highlights that remembered features in VWM are stored as episodic memory traces, and impair average estimates if these memory traces match parts of identifiable features in an ensemble display.

In addition, our results also reveal that low-level memory features of a VWM task, as a non-negligible part of scene information, have an impact on perceived high-level properties when low-level and high-level features were concurrently present. In the field of visual categorization and recognition, combining low-level features with high-level ones has been shown to have a strong effect in terms of a visual perception hierarchy (Schindler and Bartels, 2016; Stoddard et al., 2019). Accordingly, in the present study, the matching colors were irrelevant and meaningless with regards to face identity recognition, but the results indicated that these task-unrelated low-level color features were included rather than neglected or separated in VWM when participants perceive high-level face identities. From the neural mechanism at play, these low-level and high-level features are not separated from each other in the earlier visual processing task (Yang et al., 2019). Thus, low-level features cannot be ignored when predicting scene visual information where the focus is on high-level features (Gelbard-Sagiv et al., 2016; Ibarra et al., 2017). Our findings offer evidence for the influence of low-level features of VWM extending to the ensemble perceptual task. In addition, these results do not object to but rather supplement the findings of Haberman and colleagues (Haberman et al., 2015). That is, there might be a central mechanism corresponding to the observed matching feature effect in ensemble perception of both low and high-level visual features although the precision and efficiency of summary statistics were different across various levels of visual features (Haberman et al., 2015). Based on our findings, disparate features mutually affect each other when the connection of low-level properties and high-level properties is constructed in the same physical environment.

Another important detail of our study is the reconstruction between memory colors and ensemble identities. As the results of Experiment 2 mentioned, the way that features matched with the VWM object were integrated with the averaged properties in the common region, referred to as the Gestalt principle, and led to a similar common feature effect as seen in Experiment 1 which presented faces with different skin colors in an identity display. These findings are consistent with previous studies that have shown that the Gestalt principle helps combine memory stored features and ensemble properties as an integrated unit (Xu, 2002, 2006; Xu and Chun, 2007; Peterson and Berryhill, 2013; Luna et al., 2016; Kalamala et al., 2017; Montoro et al., 2017). Such integration as a whole unit is essential for the influence of memory matching features on perceptual averaging performance. It is worth noting that a smaller memory matching feature effect was found in Experiment 2 than in Experiment 1, which was found in the combined analysis. The decline in this effect might be due to the distinctly perceived integration of the ensemble display in the two experiments. Such a combination in Experiment 2 might have seemed incomplete, but the Gestalt principle seemed to play a part in classifying and connecting different features distributed across different physical spaces. Another possibility could be that less attention is spread out to the focal boundaries (i.e., on the identity faces) when a matching color is an attribute of the boxes positioned around the identity faces in the averaging task, as was done in Experiment 2. This would be in line with the findings of Nishina et al. (2007) that awareness of task-irrelevant visual features depends on the task distance, with an increase of required attention as the spatial distance between them grows.

It is worth noting that there were a few inconsistent results of the CSD and the ensemble bias in both Experiments 1 and 2. For the CSD, there was only a significant difference between CCM and MM conditions in Experiment 1 while no difference was evidenced for the rest of the comparisons in Experiments 1 and 2. Although significant differences were observed between CCM and MM conditions, the effect size (Cohen’s *d* = 0.304) of these differences was close to the effect size (Cohen’s *d* = 0.233) of differences between CM and MM conditions. Moreover, CSDs of CCM were not different from those of CM conditions. Therefore, CSD may not be a sensitive indicator to reflect the amplification effect due to matching colors in VWM. Indeed, the findings of Williams’ study (2021) also reported significant CSD differences in only one comparison (clockwise vs. control conditions) in Experiment 1. Similarly, a recent study by Iakovlev and colleagues (2021) found that the CSD did not show any difference among clockwise, counterclockwise, control conditions even when the set size increased from 4 to 16. Taken together, inconsistent CSD results between the CCM and CM conditions in our study might be due to the insensitivity of the CSD to the amplification effect. For the ensemble bias, there were mutually significant differences among CM, MM, and CCM conditions in Experiment 1. However, the difference between MM and CCM conditions became insignificant in Experiment 2 while all other differences remained significant as in Experiment 1. By taking a close look, the bias in the CCM condition did not show significant differences between Experiment 1 and 2 (*t*(70) = 0.042, *p* = 0.966, Cohen’s *d* = 0.010). However, the bias in the MM condition changed from positive in Experiment 1 to negative in Experiment 2 while both did not show a significant difference from zero (Experiment 1: *t*(35) = 1.270, *p* = 0.213, Cohen’s *d* = 0.212; Experiment 2: *t*(35) = −1.272, *p* = 0.212, Cohen’s *d* = −0.212, respectively) and reflected unbiased estimations as expected. Thus, the inconsistent result regarding MM vs. CCM comparison was possibly due to fluctuated bias in MM conditions across experiments.

## Conclusion

This study demonstrates that VWM tasks influence mean identity estimations through the memory matching color. We found that shared features learned from the VWM task were activated for the mean estimation task and impaired performance of the high-level averaging perceptual task. The impact of the VWM task on average estimates was unstable and variable, affected by the integration of the memory matching feature and perceptual averaging.

## Data availability statement

The data and experimental paradigms from all experiments are available on the Open Science Framework (https://osf.io/dc4qr/).

## Ethics statement

The studies involving human participants were reviewed and approved by the research ethics committee of Zhejiang Normal University (ZSRT2022071). The patients/participants provided their written informed consent to participate in this study.

## Author contributions

TP and JW designed the experiments and drafted the manuscript. TP contributed to the data acquisition. TP, JW, and FL contributed to the data analysis and data interpretation. TP, JW, ZZ, and FL revised the manuscript. All authors contributed to the article and approved the submitted version.

## Funding

This research was supported by the National Natural Science Foundation of Zhejiang Province of China (LY20C090003) and Open Research Fund of College of Teacher Education, Zhejiang Normal University (No. jykf21026).

## Conflict of interest

The authors declare that the research was conducted in the absence of any commercial or financial relationships that could be construed as a potential conflict of interest.

## Publisher’s note

All claims expressed in this article are solely those of the authors and do not necessarily represent those of their affiliated organizations, or those of the publisher, the editors and the reviewers. Any product that may be evaluated in this article, or claim that may be made by its manufacturer, is not guaranteed or endorsed by the publisher.
